# A Case of Bullous Pemphigoid Triggered by COVID-19 Infection in a Patient Receiving a Dipeptidyl Peptidase-4 Inhibitor

**DOI:** 10.7759/cureus.89086

**Published:** 2025-07-30

**Authors:** Mayuka Fukata, Tomomi Omura, Keiko Maeda, Takako Mori, Hiroyuki Kato, Masafumi Kuzuya

**Affiliations:** 1 Department of Residency Training, Meitetsu Hospital, Nagoya, JPN; 2 Department of Geriatric and General Internal Medicine, Meitetsu Hospital, Nagoya, JPN; 3 Department of Dermatology, Meitetsu Hospital, Nagoya, JPN

**Keywords:** autoimmune disease, covid-19, dpp4 inhibitor, drug-induced bullous pemphigoid, older patient

## Abstract

Dipeptidyl peptidase-4 (DPP-4) inhibitors are widely used for the treatment of type 2 diabetes mellitus. Recently, their association with drug-induced bullous pemphigoid (BP) has attracted increasing attention. In this report, we present a case of an 84-year-old woman who developed BP after COVID-19 infection while taking a DPP-4 inhibitor. The temporal relationship between drug administration, viral infection, and onset of autoimmune disease indicates a possible interaction between COVID-19-induced immune dysregulation and DPP-4 inhibition. The coexistence of these factors may increase the risk of developing BP in older patients with diabetes, which warrants careful monitoring.

## Introduction

Bullous pemphigoid (BP) is an autoimmune skin condition causing blisters beneath the outer layer of the skin that primarily affects individuals aged 60 and older [1]. The pathogenesis of BP involves the production of autoantibodies against BP180 and BP230, which are proteins anchoring the skin layers together [2]. Recently, the prevalence of BP has been reported to be high in Asian countries [3]. With the increasing global aging population, BP has become a more significant dermatological condition.

Dipeptidyl peptidase-4 (DPP-4) inhibitors are widely used for the treatment of type 2 diabetes mellitus. They can improve glycemic control by inhibiting the degradation of incretins, hormones that enhance insulin secretion. Since the 2010s, several studies have reported a potential association between the use of DPP-4 inhibitors and the onset of BP [4-6].

Coronavirus disease 2019 (COVID-19), which has spread globally since the end of 2019, has been reported to cause immune dysregulation, including cytokine release and T-cell activation abnormalities [7], leading to various autoimmune diseases including BP [8-11].

In this report, we present a case of an 84-year-old woman who developed BP simultaneously with COVID-19 infection and had been taking a DPP-4 inhibitor for approximately one year. The interaction between the medication and viral infection may have triggered an autoimmune response, suggesting that COVID-19 and drug use may act as a dual trigger for autoimmune disease.

## Case presentation

**Figure 1:**
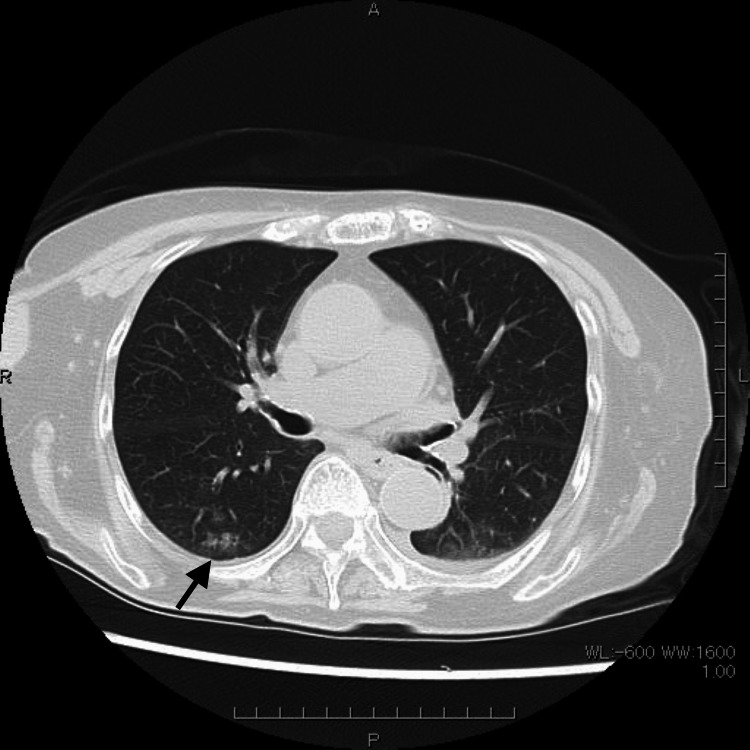
Plain chest CT Plain chest CT showing a faint area of ground-glass opacity (arrow) in the upper segment of the right lower lobe, consistent with early COVID-19 pneumonia

An 84-year-old woman presented to our outpatient clinic with fever and decreased oxygen saturation. Chest CT scan revealed pneumonia (Figure 1), and the COVID-19 antigen test was positive. Routine laboratory tests at the time of COVID-19 hospitalization are shown in Table 1A. Therefore, she was diagnosed with COVID-19 pneumonia and hospitalized. The patient had a medical history of type 2 diabetes mellitus, for which she had been receiving vildagliptin, a DPP-4 inhibitor, at a dose of 50 mg/day.

**Table 1:** Laboratory findings at hospitalization and at follow-up visit (A) Blood test results at the time of hospitalization for COVID-19. (B) Blood test results at the time of dermatology consultation, two weeks after discharge. Abbreviations: WBC, white blood cell count; TP, total protein; Alb, albumin; AST, aspartate aminotransferase; ALT, alanine aminotransferase; ALP, alkaline phosphatase; LDH, lactate dehydrogenase; Na, sodium; K, potassium; Cl, chloride; BUN, blood urea nitrogen; Cr, creatinine; Glu, glucose; HbA1c (NGSP), hemoglobin A1c (NGSP); anti-Dsg1 antibody, anti-desmoglein 1 antibody; anti-Dsg3 antibody, anti-desmoglein 3 antibody; n.d., not determined/no data available.

Component	Units	Reference Range	Results	
			A	B
WBC	/μl	2.2-8.6x10^3^	8.48x10^3^	9.00x10^3^
Neutrophils	%	40-74	85	72.2
Eosinophils	%	1-9	1	4.7
Lymphocytes	%	20-49	7	18.2
Monocytes	%	2-9	6	4.7
Basophils	%	0-1	1	0.2
Hemoglobin	g/dL	11.6-14.8	11.4	12.9
Platelets	/μl	158-348x10^3^	282x10^3^	282x10^3^
TP	g/dL	6.6-8.1	6.2	6.3
Alb	g/dL	4.1-5.1	2.8	n.d.
AST	U/L	13-30	13	17
ALT	U/L	7-23	8	10
ALP	U/L	38-113	90	97
LDH	U/L	124-222	146	196
BUN	mg/dL	8-20	8	9
Cr	mg/dL	0.46-0.79	0.61	0.63
Na	mmol/L	138-145	137	139
K	mmol/L	3.6-4.8	4.3	4.5
Cl	mmol/L	101-108	103	104
CRP	mg/dL	0.00-0.14	2.89	1.22
Glu	mg/dL	73-109	152	n.d.
HbA1c	%	4.9-6.0	6.8	n.d.
SARS-COV-2 antigen			Positive	
Total IgE	IU/mL	~173	n.d.	585
Anti-BP180 antibody	U/mL	~8.9	n.d.	23.8
Anti-Dsg1 antibody	U/mL	~19	n.d.	<3.0
Anti-Dsg3 antibody	U/mL	~19	n.d.	<3.0

**Figure 2:**
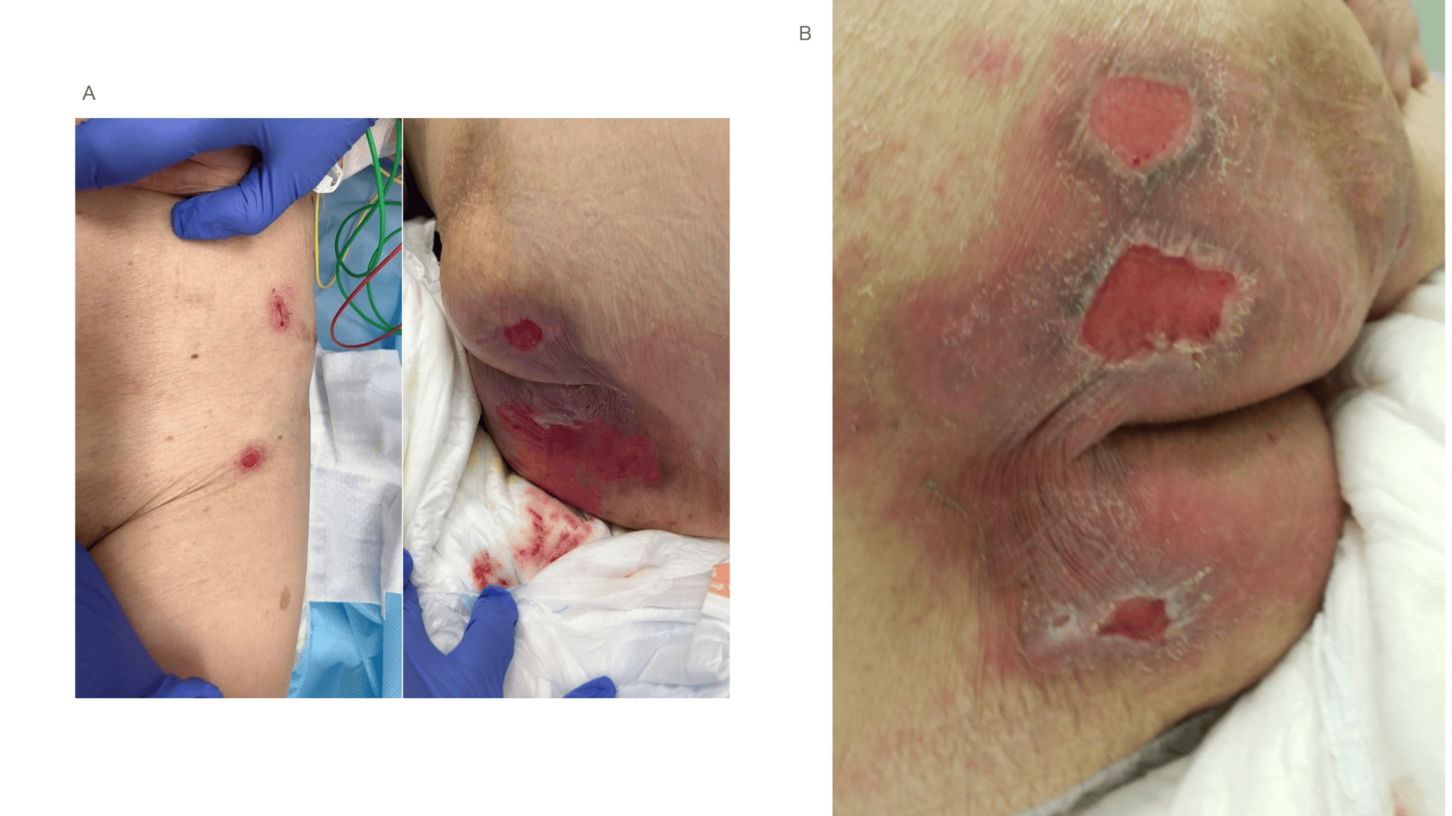
Erosions of the thigh and buttocks during hospitalization and at outpatient follow-up (A) Erosions with surrounding erythema observed on the left thigh (left panel) and bilateral buttocks (right panel) on the day of admission for COVID-19. (B) Worsening erosions on the buttocks observed two weeks after discharge, prior to the initiation of corticosteroid therapy. The lesions were intensely pruritic. No intact bullae were observed at this time.

On admission, the patient developed erosions with severe pruritus in the left inguinal and bilateral gluteal regions, showing an asymmetrical distribution (Figure 2A). Although the erosions were accompanied by surrounding erythema, no tense bullae or urticarial erythema were noted. Neither the patient nor her family had noticed any skin findings other than intense itching and erosions. The patient was treated with remdesivir and dexamethasone for COVID-19 pneumonia. The skin erosions were initially considered to be pressure ulcers and were treated with zinc oxide ointment and betamethasone ointment. Her pneumonia improved, and no new erosions appeared. She was discharged on hospital day 19.

However, two weeks after discharge, the patient visited our dermatology department due to worsening of the erosions and intense pruritus (Figure 2B). The family reported that blisters had developed and were subsequently scratched. No mucosal lesions were observed, and the Nikolsky sign was negative. Although intact blisters were not observed due to excoriation from severe pruritus, the presence of intense itching and the family's report of blisters led to the suspicion of BP. Anti-BP180 antibody testing revealed an elevated level of 23.8 U/ml (reference range: ~8.9 U/mL; diagnostic cutoff: ≥9.0 U/mL), which was presumed to correspond to the NC16A domain, as commonly measured in standard clinical settings. Other blood test data, shown in Table 1B, were C-reactive protein (CRP) at 1.22 mg/dl (reference range: ~0.14 mg/dl), white blood cells (WBC) at 9.00x10^3^ /μl (reference range: 2.2-8.6x10^3^ /μl), eosinophils at 4.7 % (reference range: 1%-9 %), and total IgE at 585 IU/ml (reference range: ~173 IU/mL). A skin biopsy was not performed due to excoriation of the lesions, which made it difficult to identify an appropriate biopsy site, and because the patient and her family declined the procedure. Although no skin biopsy was performed, the patient was clinically diagnosed with BP. Treatment with oral prednisolone at 30 mg/day was initiated, and no additional erosions were observed.

Vildagliptin was continued because the initial skin lesions were presumed to be pressure ulcers, and even after the diagnosis of BP, the medication was not discontinued due to a lack of communication between the hospital and the primary care physician. The drug was eventually discontinued one month later. Prednisolone was gradually tapered to 15 mg/day, and no recurrence of skin lesions has been observed to date.

## Discussion

BP is an autoimmune blistering disease that predominantly affects older adults. BP is characterized by the production of autoantibodies against BP180 and BP230, which are components of the epidermal basement membrane. These autoantibodies bind to the basement membrane, causing skin damage. Skin biopsy to identify subepidermal blisters and direct immunofluorescence to detect immunoglobulin deposition are valuable diagnostic tools for BP.

In our case, erosions with intense pruritus appeared in the buttocks and inguinal regions on the same day as the onset of COVID-19. There were no newly initiated systemic or topical medications before the onset of erosions, making other drug eruptions or contact dermatitis unlikely. Initially, the skin lesions were considered pressure ulcers and were treated with betamethasone ointment and zinc oxide ointment, resulting in prompt improvement. However, the lesions worsened after discharge. Although no healthcare providers observed the blisters, the patient’s family reported the appearance of blisters that were subsequently scratched, leading to suspicion of BP. Testing revealed positive anti-BP180 antibodies. Although a skin biopsy could not be performed in our case, the diagnosis of BP was made clinically based on the elevated anti-BP180 antibody titer, and the cutaneous symptoms and pruritus improved after the initiation of oral prednisolone. In this case, the anti-BP180 antibody titer was 23.8 U/mL, which represents a mild elevation above the diagnostic cutoff of 9 U/mL. Although this level is not markedly high, it is considered clinically meaningful in the context of compatible symptoms. It has also been reported that some diabetic patients taking DPP-4 inhibitors may test positive for anti-BP180 antibodies in the absence of clinical symptoms [12], highlighting the importance of correlating serologic findings with clinical presentation. It is difficult to completely exclude other erosive dermatoses such as irritant dermatitis, drug eruption, or viral exanthems. However, based on the presence of intense pruritus, the family's report of blister formation, a positive anti-BP180 antibody test, and the clinical response to corticosteroid therapy, BP was considered the most consistent diagnosis.

DPP-4 inhibitors have been reported to be associated with the onset of BP [4-6]. DPP-4, also known as the T-cell surface antigen CD26, plays a crucial role in the immune system [13]. It influences the Th1/Th2 balance, production of inflammatory cytokines, and activation of immune cells, such as T and B cells [14]. Furthermore, DPP-4 has been reported to enhance eosinophil migration into the dermis via eotaxin [15]. The mechanism by which DPP-4 inhibitors induce BP remains unclear. However, such immune alterations may contribute to the pathogenesis of BP. The persistent use of DPP-4 inhibitors has been reported to precipitate the exacerbation of BP. Therefore, discontinuing their use without delay is strongly advised. A revised Naranjo score of 4 was calculated, based on the temporal relationship, known adverse effect profile, clinical improvement after drug discontinuation, and the absence of alternative definitive causes. This score suggests a “possible” adverse drug reaction [16]. According to the WHO-Uppsala Monitoring Centre (UMC) system for standardized case causality assessment, the relationship between vildagliptin and BP in this case would also be classified as "possible." This classification reflects a plausible temporal relationship, lack of a definitive alternative cause, and limited dechallenge information [16]. Additionally, in this case, vildagliptin was not discontinued immediately after the diagnosis of BP due to a communication lapse between our hospital and the patient’s primary care provider. This highlights the importance of timely information-sharing and collaboration among healthcare providers, particularly when managing adverse drug reactions. Improved communication among health care providers could have led to earlier drug discontinuation and optimized patient care.

COVID-19 infection can cause systemic immune dysregulation, including excessive production of inflammatory cytokines, such as interleukin (IL)-17, tumor necrosis factor (TNF)-α, and IL-1β, and regulatory T-cell dysfunction [7]. These immunologic changes may enhance the pathogenic reactivity of autoantibodies, thereby contributing to the onset of autoimmune diseases.

Only two cases of BP (Table 2) associated with COVID-19 infection and DPP-4 inhibitor use have been reported. In the first case, BP developed approximately two months after COVID-19 infection, after which treatment with DPP-4 inhibitors was initiated during remission. Subsequently, the patient developed severe recurrence of BP, which was considered to be due to the synergistic effect of COVID-19 infection and DPP-4 inhibitors [17]. In this case, BP developed after COVID-19 infection and recurred after the initiation of DPP-4 inhibitor treatment. Therefore, the causal relationship between COVID-19 infection and DPP-4 inhibitors and the onset of BP remains unclear. In the other case reported by Fujita et al., the patient had received a DPP-4 inhibitor for 1.5 years more than a decade prior to the onset of BP, and developed BP four days after contracting COVID-19 [18]. Although the timing of COVID-19 infection and BP onset differs from our case, this case is similar in that the patient had taken DPP-4 inhibitors for a prolonged period, and BP appeared early after COVID-19 infection.

**Table 2:** Clinical comparison of reported cases of bullous pemphigoid (BP) associated with COVID-19 and dipeptidyl peptidase-4 (DPP-4) inhibitor use Comparison of the present case with two previously reported cases of BP associated with COVID-19 infection and DPP-4 inhibitor use. Key clinical features, medication history, timing of BP onset relative to COVID-19 infection, laboratory findings, and treatment outcomes are summarized. BP180 antibody subtype (NC16A or full-length) is noted when available. All cases, except the present one, were confirmed by skin biopsy. Abbreviations: PSL, prednisolone.

Case	Age / Sex	DPP-4 Inhibitor (Agent, Timing)	COVID-19 Infection	BP Onset Timing	Anti-BP180 Antibody(U/ml)	Skin Biopsy	Treatment	Outcome / Recurrence
1 (Ref. [17])	73 / M	Teneligliptin, started Feb 2021 (after initial BP onset)	Dec 2020	Feb 2021 (2 months after COVID-19); recurrence in Apr 2022 after steroid discontinuation	Not described	Performed	Initial: deflazacort, dapsone, systemic steroid; Recurrence: steroid pulse therapy → deflazacort, dapsone, azathioprine, nicotinamide	Improved after recurrence treatment
2 (Ref. [18])	74 / M	Vildagliptin, taken for 1.5 years (over 10 years ago)	COVID-19; BP onset 4 days later	Day 4 after infection	Initial: NC16A (-), Full-length BP180: 170.48 10 weeks later: NC16A: 47.5	Performed	PSL 20mg /day, mycophenolate mofetil	Not described
Present Case	84 / F	Vildagliptin, taken for 1 year (ongoing at BP onset)	Diagnosed on admission	Same day as COVID-19 diagnosis	NC16A: 23.8	Not performed	PSL 30 mg/day; DPP-4 inhibitor discontinued after 1 month	No recurrence after steroid treatment

The findings of these two cases indicate that COVID-19 and DPP-4 inhibitors may interact and contribute to the development of BP. In our present case and the case reported by Fujita et al., erosions developed around the time of confirmation of a positive COVID-19 infection diagnosis, which was likely too early for an antibody-mediated immune response to have developed, as noted by Fujita et al. Furthermore, a subset of diabetic patients taking DPP-4 inhibitors, even without BP symptoms, have been reported to test positive for anti-BP180 antibodies [12].

These findings suggest that, rather than inducing new autoantibody production, the COVID-19 infection may serve as a "final trigger" in patients with a chronically altered immune state resulting from DPP-4 inhibitor therapy, leading to the pathological activation of pre-existing autoantibodies. The presence of autoantibodies alone may not directly lead to disease onset. A change in the immune environment or the presence of an external trigger may be necessary for clinical manifestation.

## Conclusions

This case suggests that the onset of an autoimmune disease may have been triggered by a "dual-trigger" mechanism involving chronic immune modulation by a DPP-4 inhibitor and acute immune stimulation from COVID-19 infection. Clinicians should consider the potential risk of BP in patients with COVID-19 receiving DPP-4 inhibitors. This case adds to a small but growing body of evidence suggesting that clinicians should be vigilant for BP in older diabetic patients with COVID-19 receiving DPP-4 inhibitors. However, one important limitation of this case is that a skin biopsy was not performed, and therefore a definitive diagnosis could not be established. This limitation should be taken into consideration when interpreting the findings.
